# Correction to: Suppressive effects of plumbagin on invasion and migration of breast cancer cells via the inhibition of STAT3 signaling and down-regulation of inflammatory cytokine expressions

**DOI:** 10.1038/s41413-019-0052-0

**Published:** 2019-05-22

**Authors:** Wei Yan, Bing Tu, Yun-yun Liu, Ting-yu Wang, Han Qiao, Zan-jing Zhai, Hao-wei Li, Ting-ting Tang

**Affiliations:** 1 Shanghai Key Laboratory of Orthopaedic Implants, Department of Orthopaedic Surgery, Shanghai Ninth People’s Hospital, Shanghai Jiao Tong University School of Medicine, Shanghai, 200011 China; 2 Wendeng Zhenggu Hospital of Shandong Province, Wendeng, Shandong 264400 China; 3 Department of Gynecology and Obstetrics, Wendeng Center Hospital of Weihai City, Weihai, Shandong 264400 China; 4 Department of Pharmacy, Shanghai Ninth People’s Hospital, Shanghai Jiao Tong University School of Medicine, Shanghai, 200011 China

**Correction to: Bone Research** (2013) **1**:362–370; 10.4248/BR201304007; published online 31 December 2013.

During re-read of our previously article [1] published in Bone Research, we regretted to find a mistake in Fig. 1B. The representative figure chosen to compare the inhibitory effect of 0 μmol·L^−1^ plumbagin upon breast cancer metastasis was wrong due to the mishandling in manuscript preparation. Although this correction does not affect the results and conclusion of above paper, all the authors agree to correct this negligence as providing the right Fig. 1 presented below. We feel sorry and apologize for all the inconvenience it caused.

**Figure 1:**
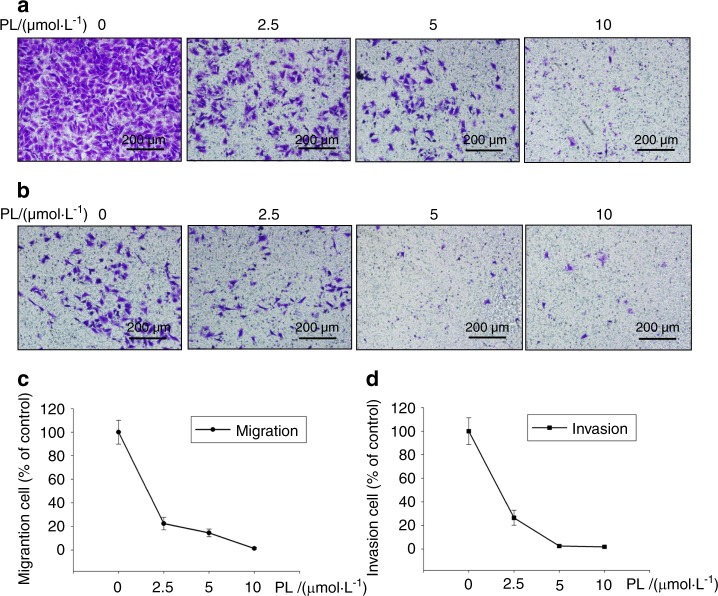
Fig. 1

1. Yan, W. et al. Suppressive effects of plumbagin on invasion and migration of breast cancer cells via the inhibition of STAT3 signaling and downregulation of inflammatory cytokine expressions. *Bone Res.* **4**, 362–370 (2013).

